# Optimization reconstruction of projective point of laser line coordinated by orthogonal reference

**DOI:** 10.1038/s41598-017-15399-1

**Published:** 2017-11-07

**Authors:** Guan Xu, Jing Yuan, Xiaotao Li, Jian Su

**Affiliations:** 1 Traffic and Transportation College, Nanling Campus, Jilin University, Renmin Str. 5988#, Changchun, China; 2 School of Mechanical Science and Engineering, Nanling Campus, Jilin University, Renmin Str. 5988#, Changchun, China

## Abstract

A 3D reconstruction method is presented for the laser projective point of a laser line, which is located by an orthogonal reference. The laser line is initially expressed by the Plücker matrix generated from two random points on the line and then transformed to the dual Plücker matrix representation. The initial solution of the 3D laser point is obtained by the non-homogeneous linear equations, which are derived from the projection geometry of the 3D feature point on the reference and the 3D laser point on the laser line represented by the dual Plücker matrix. The optimization function is constructed by minimizing the sums of the re-projection errors of the reference points and the laser point. The average absolute error of the initial solution is 1.07 mm while the one of the optimization solution is 1.01 mm. The average relative error of the initial solution is 4.14% while the one of the optimization solution is 3.86%. Thus, the optimization reconstruction of the projective point contributes the accuracy and the prospect in the vision-based inspection fields.

## Introduction

Active vision with the structured light is considered as one of the most promising approach for the optical detection and profile reconstruction, where the line-structured light is attractive due to the high accuracy and efficiency^1–8^. In the vision-based measurement, the calibration methods with the 1D references^9,10^, 2D references^11–16^ or 3D references^17–19^ are previously studied to construct the projection geometry of a camera in the measurement. The 1D-reference-based calibration was proposed by Zhang^9^ for the first time. In the calibration process, the collinear feature points with the known distances between them are selected on the reference. The 1D-reference-based calibration is performed by rotating the reference around a fixed point. Peng^10^ states a novel approach to calibrate the camera, which only requires a very simple object with two end points. The extrinsic and intrinsic camera parameters are obtained from the poses of 1D target in different positions. The robotic calibration with 2D reference is provided by Zhang^11^. The homographs from the 2D world points on the reference to the 2D image points are solved by the homogeneous linear equations, firstly. Then, the camera parameters are decomposed from the orthogonality condition of the rotation matrix. Xu^12^ introduces a simple calibration method by using the Plücker matrices with the help of the 2D target images. The laser plane is built with the Plücker matrices of the intersection lines between the target plane and the laser plane. An improved calibration method on the basis of the perpendicularity compensation is introduced by Jia^13^ for the binocular vision measurement system. The perpendicularity compensation is adopted to enhance the calibration accuracy. Lv^14^ proposes the pinhole camera pattern with the lens distortion and a reliable method by utilizing the random sample consensus algorithm to automatically select a subset of the calibration images to evaluate camera parameters. Fathi^15^ proposes a conversion method to calibrate the camera for the application in the 3D reconstruction. Traditional calibration algorithms are used for the multistep procedures to obtain the parameters optimized for a particular calibration depth. The parameter sets are finally used for the beam adjustment steps to obtain the 3D coordinates. Zhang^16^ provides a flexible 3D calibration method by a white board with the hollow ring markers. The intrinsic camera parameters are calibrated by the extracted positions of the markers.

The 1D and 2D references are easier to be manufactured and used than the 3D reference. The 1D reference method adopts at least six images to perform the camera calibration. However, the calibration precision of the 1D reference is usually lower than the 2D and 3D references due to the lack of the calibration information. Therefore, camera calibration approaches are mainly based on the 2D or 3D references. The 2D reference method realizes the calibration by three or more images. Furthermore, the 3D reference method achieves the calibration only by one image, which is the obviously advantage of the method. The 3D reference method is mentioned in the literature of Abdel-Aziz^17^. The camera parameters are solved by the homograph from the 3D points on the reference to the 2D points on the image. A new camera calibration method of the laser plane is presented by Xu^18^ with a 3D reference and a 1D reference. The bottom of the 1D reference is a cone and the top of it is marked with a checkerboard pattern. The 2D coordinates of the intersection points between the laser plane and the 1D reference are extracted for the laser plane calibration. A calibration method is provided by Léandry^19^ with a 3D calibration reference. A calibration procedure on the basis of polynomial transformations is presented for the structured-light projection. The measurement of a large object in a short distance is discussed in the experiments. In order to reconstruct the laser projection point from one image, a laser projector with an orthogonal 3D reference, the bridge from the camera to the laser line, is designed to reconstruct the projective point of the laser line. Considering the 3D laser line can be denoted by the Plücker matrix directly, the 3D reconstruction of the projective laser point is performed by employing the Plücker matrix of the laser line.

The following paper is outlined as follows: Section 2 illustrates the reconstruction model. The initial solution and the optimization solution of the laser projective point are also introduced in this section. Section 3, the experiments are performed to verify the reconstruction method and the displacement is considered as the evaluation benchmark in this part. Moreover, the absolute and relative errors are obtained to evaluate the reconstruction method. We conclude with a summary in Section 4.

## Methods

**Figure 1:**
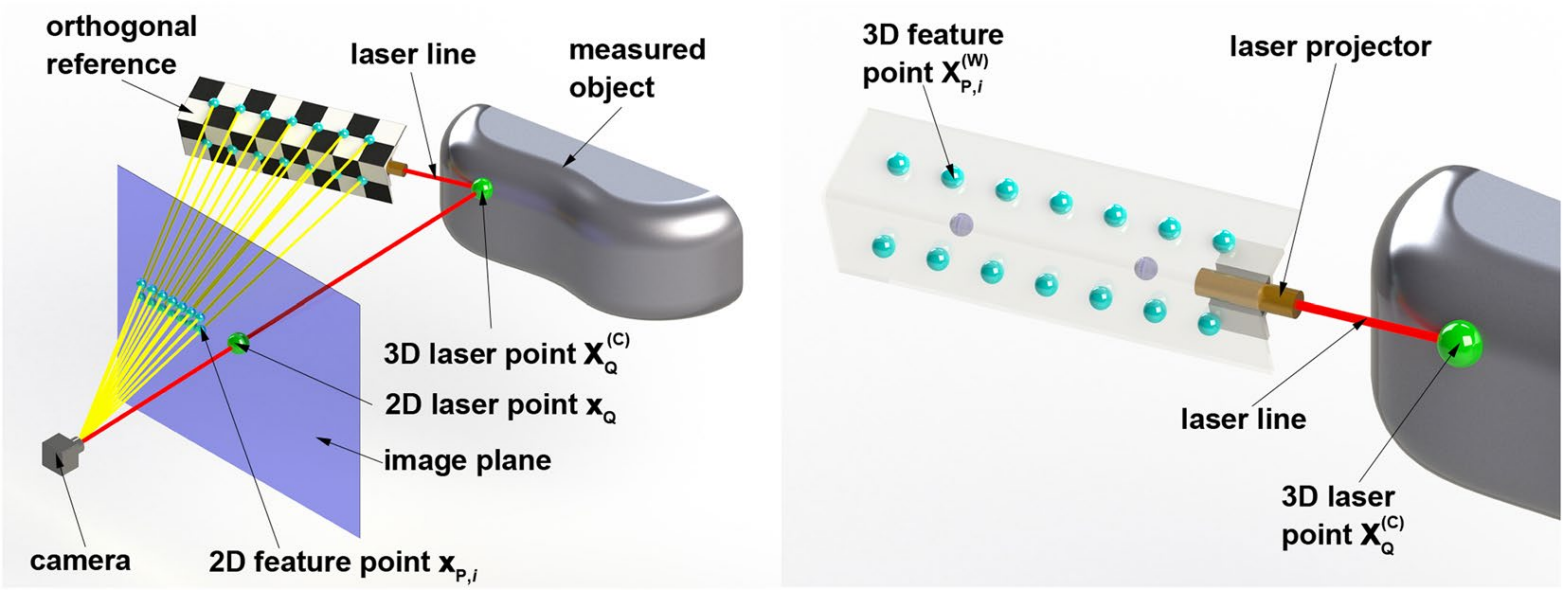
Reconstruction approach of the laser projection point that is coordinated by the orthogonal reference (see Supplementary Video S1). (**a**) Reconstruction principle. (**b**) Enlarged and transparent details of the reconstruction instruments.

As illustrated in Fig. 1, the reconstruction system mainly includes a camera and an orthogonal reference with a laser projector. The 3D feature points are derived from the check board pattern on the orthogonal reference. The laser line is projected on the measured object. The 3D laser point on the measured object and the 3D feature points are captured in the image plane of the camera. The world coordinate system *o*-*xyz* is defined by the orthogonal reference. The image coordinate system is determined by the captured image.

For a 3D point **X** in the world coordinate system, the 2D projective point **x** in the captured image is expressed by^20,21^

1 $${\bf{x}}={\rm{P}}{\bf{X}}$$

where P = A_CI_[R_WC_ **t**_WC_] is the transform matrix from the world coordinate system to the image coordinate system and calibrated by the 3D reference^20^. A_CI_ = [A_*ij*_]_3×3_ is the intrinsic matrix of the camera. R_WC_, **t**_WC_ are the rotation matrix and translation vector of the camera about the world coordinate system, respectively.

As the distances from the laser line to the two orthogonal reference planes are both identical to the radius *r* of the cylindrical laser projector, two virtual points on the laser line are ${{\bf{X}}}_{{\rm{A}}}^{({\rm{W}})}={(r,{\lambda }_{1},r,1)}^{T}$, ${{\bf{X}}}_{{\rm{B}}}^{({\rm{W}})}={(r,{\lambda }_{2},r,1)}^{{\rm{T}}}$, where *λ*_1_, *λ*_2_ are the point distances along the *o*-*y* axis of the world coordinate system and denoted by two random real numbers.

The two points ${{\bf{X}}}_{{\rm{A}}}^{({\rm{W}})}$, ${{\bf{X}}}_{{\rm{B}}}^{({\rm{W}})}$ in the world coordinate system are expressed in the camera coordinate system by^20^

2 $${{\bf{X}}}_{{\rm{A}}}^{({\rm{C}})}=[{{\bf{R}}}_{{\rm{WC}}}{{\bf{t}}}_{{\rm{WC}}}]{{\bf{X}}}_{{\rm{A}}}^{({\rm{W}})}$$

3 $${{\bf{X}}}_{{\rm{B}}}^{({\rm{C}})}=[{{\bf{R}}}_{{\rm{WC}}}{{\bf{t}}}_{{\rm{WC}}}]{{\bf{X}}}_{{\rm{B}}}^{({\rm{W}})}$$

The laser line that passes through the two points ${{\bf{X}}}_{{\rm{A}}}^{({\rm{W}})}$, ${{\bf{X}}}_{{\rm{B}}}^{({\rm{W}})}$ is

4 $${{\bf{L}}}^{({\rm{C}})}={{\rm{X}}}_{{\rm{A}}}^{({\rm{C}})}{{\rm{X}}}_{{\rm{B}}}^{({\rm{C}}){\rm{T}}}-{{\rm{X}}}_{{\rm{B}}}^{({\rm{C}})}{{\rm{X}}}_{{\rm{A}}}^{({\rm{C}}){\rm{T}}}$$

where L^(C)^ = [*l*_*ij*_]_4×4_ is the Plücker matrix of the intersection laser line in the camera coordinate system.

Then, the dual Plücker matrix of the laser line L^(C)*^ = [${l}_{ij}^{\ast }$]_4×4_ denoted by two intersecting planes is obtained from the Plücker matrix of the laser line expressed by the two points, according to the relationship of ${{l}}_{34}^{\ast }:{{l}}_{42}^{\ast }:{{l}}_{23}^{\ast }:{l}_{14}^{\ast }:{{l}}_{13}^{\ast }:{{l}}_{12}^{\ast }={l}_{12}:{l}_{13}:{l}_{14}:{l}_{23}:{l}_{42}:{l}_{34}$ ^20^. As the 3D laser point is located on the laser line L^(C)*^, we have

5 $${{\rm{L}}}^{({\rm{C}})\ast }{{\bf{X}}}_{{\rm{Q}}}^{({\rm{C}})}={\bf{0}}$$

where ${{\rm{X}}}_{{\rm{Q}}}^{({\rm{C}})}={[{{\rm{X}}}_{{\rm{Qx}}}^{({\rm{C}})},{{\rm{X}}}_{{\rm{Qy}}}^{({\rm{C}})},{{\rm{X}}}_{{\rm{Qz}}}^{({\rm{C}})},1]}^{{\rm{T}}}$ is the 3D laser point on the laser line L^(C)*^, **0** is a zero vector.

The pin-hole-model^20^ projection from the 3D laser point ${{\bf{X}}}_{{\rm{Q}}}^{({\rm{C}})}$ to the 2D point **x**_Q_ = [**x**_Q*x*_, **x**_Q*y*_, 1]^T^ in the image is described by

6 $${{\bf{A}}}_{{\rm{CI}}}{{\bf{X}}}_{{\rm{Q}}}^{({\rm{C}})}={{\bf{x}}}_{{\rm{Q}}}$$

Stacking Eqs (5) and (6), then

7 $${\rm{B}}{{\bf{X}}}_{{\rm{Q}}}^{({\rm{C}})}={\bf{C}}$$

where $${\rm{B}}=[\begin{array}{cccc}{A}_{11} & {A}_{12} & {A}_{13} & 0\\ 0 & {A}_{22} & {A}_{23} & 0\\ 0 & 0 & 1 & 0\\ 0 & {l}_{12}^{\ast } & {l}_{13}^{\ast } & {l}_{14}^{\ast }\\ -{l}_{12}^{\ast } & 0 & {l}_{23}^{\ast } & -{l}_{42}^{\ast }\\ -{l}_{13}^{\ast } & -{l}_{23}^{\ast } & 0 & {l}_{34}\\ -{l}_{14}^{\ast } & {l}_{42}^{\ast } & -{l}_{34}^{\ast } & 0\end{array}],{\bf{C}}=[\begin{array}{c}{{\bf{x}}}_{{\rm{Q}}{\rm{x}}}\\ {{\bf{x}}}_{{\rm{Q}}{\rm{y}}}\\ 1\\ 0\\ 0\\ 0\\ 0\end{array}]$$

The solution of the non-homogeneous linear equations in Eq. (7) is

8 $${{\bf{X}}}_{{\rm{Q}}}^{({\rm{C}})}={{({\rm{B}}}^{{\rm{T}}}{\rm{B}})}^{-1}{{\rm{B}}}^{{\rm{T}}}{\rm{C}}$$

The extracted 2D points in the image are often affected by the noises; therefore, the re-projection errors of the 3D laser point ${{\bf{X}}}_{{\rm{Q}}}^{({\rm{C}})}$ in the camera coordinate system and the 3D feature points ${{\bf{X}}}_{{\rm{P}},i}^{({\rm{W}})}$ in the world coordinate system are parameterized by the 3D laser point ${{\bf{X}}}_{{\rm{Q}}}^{({\rm{C}})}$, the intrinsic matrix A_CI_, the rotation matrix R_WC_ and the translation vector **t**_WC_. The initial solutions are then optimized by

9 $$\{{{\bf{X}}}_{{\rm{Q}}}^{({\rm{C}})},{{\rm{A}}}_{{\rm{CI}}},{{\rm{R}}}_{{\rm{WC}}},{{\bf{t}}}_{{\rm{WC}}}\}=\text{arg}\,\min \{{\Vert {{\rm{A}}}_{{\rm{CI}}}{{\bf{X}}}_{{\rm{Q}}}^{({\rm{C}})}-{{\bf{x}}}_{{\rm{Q}}}\Vert }^{2}+\sum _{i=1}^{N}{\Vert {{\rm{A}}}_{{\rm{CI}}}[{{\rm{R}}}_{{\rm{WC}}}{{\bf{t}}}_{{\rm{WC}}}]{{\bf{X}}}_{{\rm{P}},i}^{({\rm{W}})}-{{\bf{x}}}_{{\rm{P}},i}\Vert }^{2}\}$$

where ${{\bf{X}}}_{{\rm{P}},i}^{({\rm{W}})}$ is the *i*th 3D feature point in the world coordinate system, **x**_P,*i*_ is the *i*th 2D projective point of the *i*th 3D feature point, *N* is the number of the feature points.

## Results

The image resolution of 2048 × 1536 is chosen for the experiments. The orthogonal reference includes two 300 mm × 60 mm planes. Each plane of the reference is covered by 2 × 10 feature points. The initial solutions of 3D laser points are compared with the optimized solutions in two groups of experiments.

**Figure 2:**
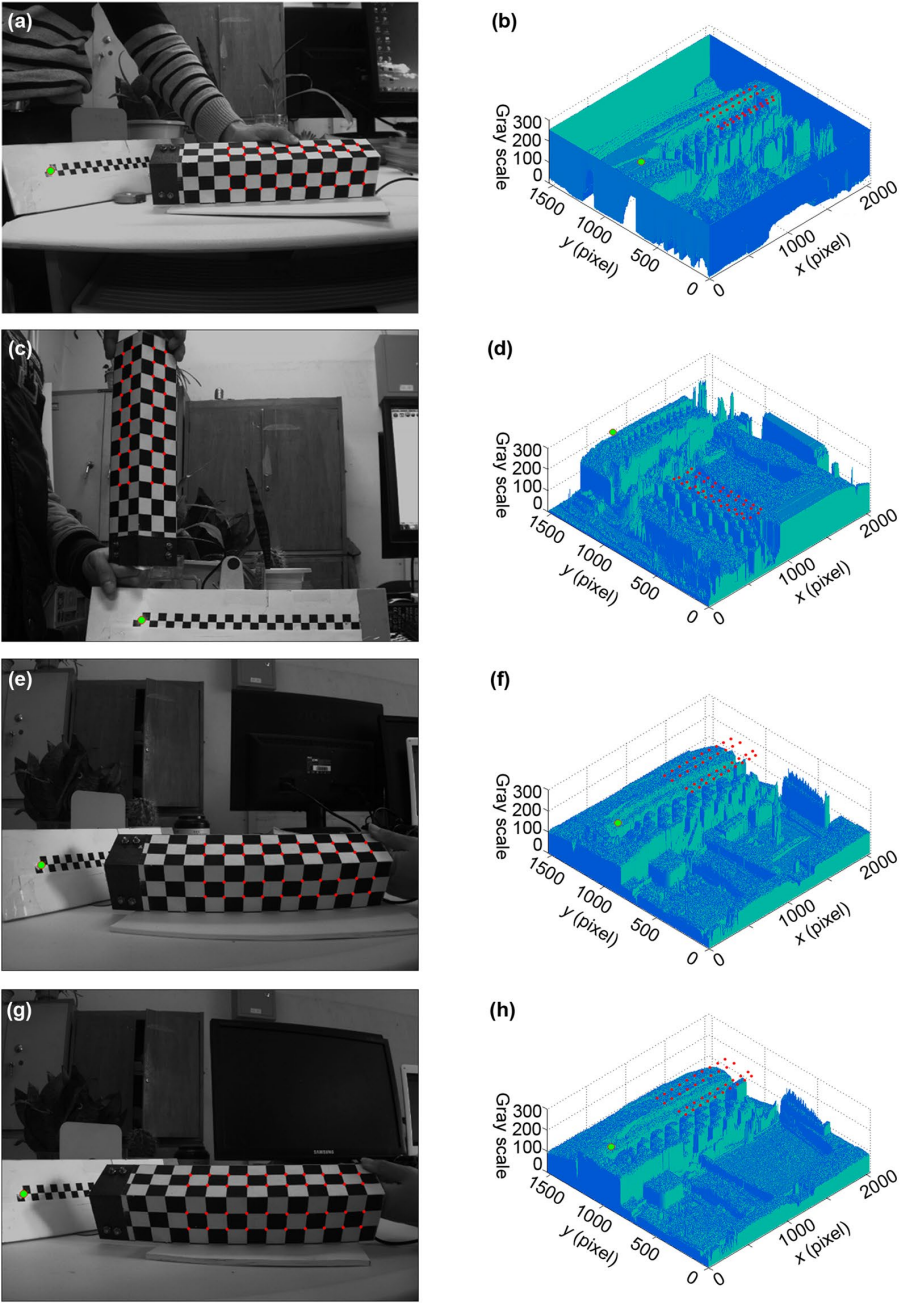
Results of extracted laser points and feature points. (**a**) Extracted image points in the first group of experiments. (**b**) Grey-scale map in the first group of experiments. (**c**) Extracted image points in the second group of experiments. (**d**) Grey-scale map in the second group of experiments. (**e**) Extracted image points in the third group of experiments. (**f**) Grey-scale map in the third group of experiments. (**g**) Extracted image points in the fourth group of experiments. (**h**) Grey-scale map in the fourth group of experiments.

The projective laser point and the projective feature points are extracted from the image and drawn in the grey-scale map, as shown in Fig. 2. The measurement aims to reconstruct the 3D coordinates of the projective point of the laser line coordinated by the orthogonal reference. Moreover, the reconstructed 3D points are defined in the camera coordinate system, whose axis benchmarks and origin benchmark are the virtual geometrical elements. Therefore, the relative benchmark is chosen to evaluate the reconstruction accuracy. A planar ruler with the check board pattern is employed for the evaluation. The displacement between two feature points on the ruler is considered as the relative benchmark. In order to evaluate the initial solutions and the optimization solutions of the 3D laser points, the 3D laser points are projected to the feature points on the planar ruler with the check board pattern. The 3D coordinates of the laser points are reconstructed by this method. The reconstructed displacement between two 3D laser points is contributed from the norm of the difference between the reconstructed coordinates of the two 3D laser points. The benchmark displacement between two feature points can be provided from the planar ruler. Accordingly, the reconstruction error of the displacement is obtained from the difference between the real displacement on the ruler and the reconstructed displacement.

Four groups of experiments are performed to test the absolute errors and relative errors of the reconstruction method. The absolute and relative errors of the reconstruction displacement are defined by^22^

10 $${\rm{\Delta }}d=|d-{d}_{0}|$$

11 $$E=|d-{d}_{0}|/{d}_{0}$$

where Δ*d* is the absolute error. *E* is the relative error. *d*_0_ is the benchmark displacement between two feature points on the ruler. *d* is the reconstructed displacement between two laser points.

**Figure 3:**
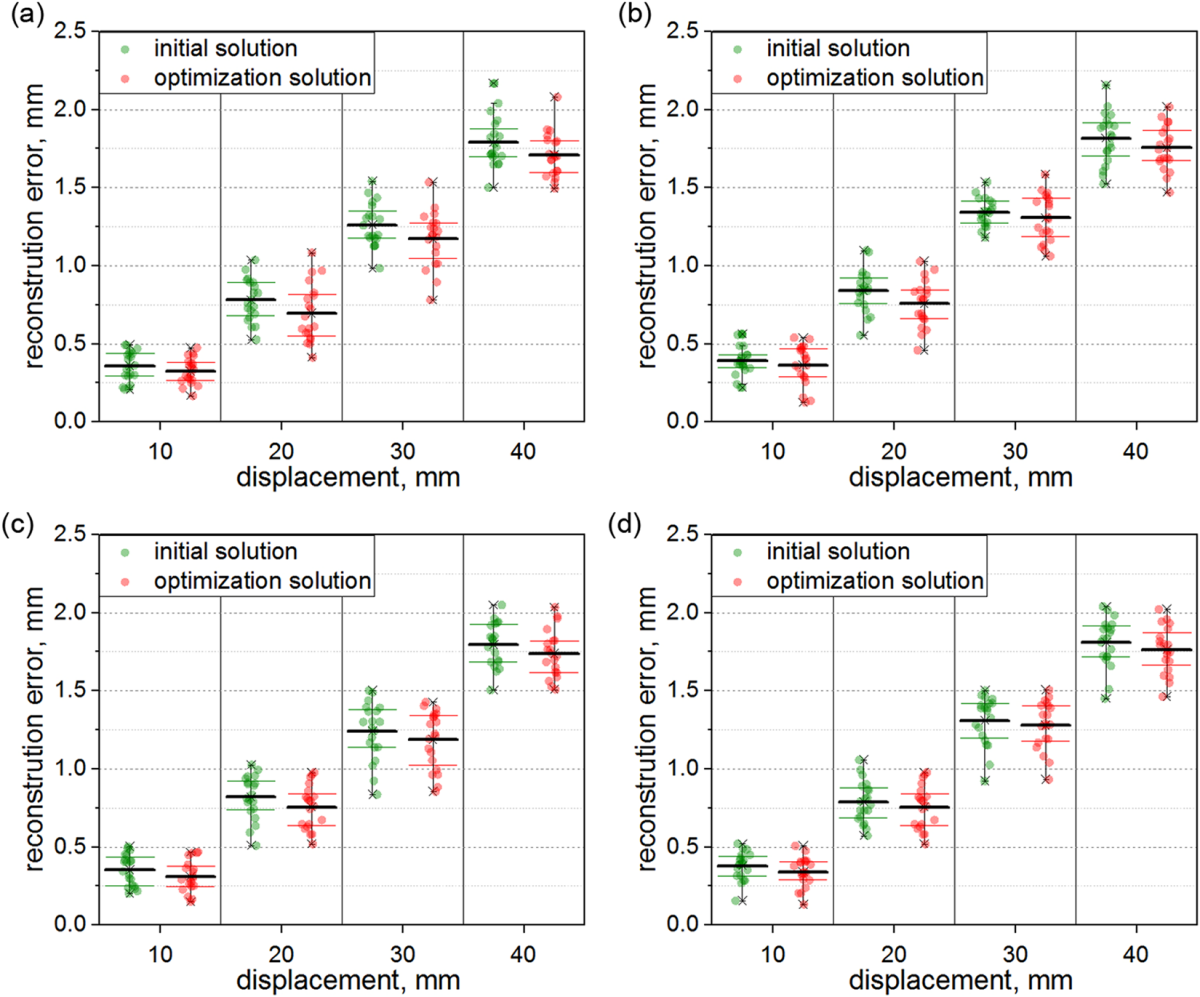
Absolute errors of the reconstruction displacements, 10 mm, 20 mm, 30 mm, 40 mm. (**a**) The first group of experiments. (**b**) The second group of experiments. (**c**) The third group of experiments. (**d**) The fourth group of experiments.

The absolute errors of the reconstruction displacements are shown in Fig. 3. The black lines represent the mean errors at different displacements. In the first group of experiments, the error means of the initial solution and the optimization solution are 0.36 mm and 0.32 mm with the displacement of 10 mm. Then, when the displacement increases to 20 mm, the average reconstruction errors of the initial solution and the optimization solution are 0.78 mm and 0.70 mm. The error means of the initial solution and the optimization solution are 1.26 mm and 1.17 mm with the displacement of 30 mm. Finally, when the displacement grows to 40 mm, the average reconstruction errors of the initial solution and the optimization solution are 1.79 mm and 1.71 mm. In the second group of experiments, the error means of the initial solution and the optimization solution are 0.39 mm and 0.36 mm with the displacement of 10 mm. Then, when the displacement rises to 20 mm, the average reconstruction errors of the initial solution and the optimization solution are 0.84 mm and 0.76 mm. The error means in the initial method and the optimization method are 1.34 mm and 1.31 mm with the displacement of 30 mm. Finally, when the displacement grows to 40 mm, the average reconstruction errors in the initial solution and the optimization solution are 1.82 mm and 1.75 mm. In the third group of experiments, the error means of the initial solution and the optimization solution are 0.35 mm and 0.31 mm with the displacement of 10 mm. Then, when the displacement rises to 20 mm, the average reconstruction errors of the initial solution and the optimization solution are 0.82 mm and 0.75 mm. The error means in the initial method and the optimization method are 1.24 mm and 1.19 mm with the displacement of 30 mm. Finally, when the displacement grows to 40 mm, the average reconstruction errors in the initial solution and the optimization solution are 1.80 mm and 1.74 mm. In the fourth group of experiments, the error means of the initial solution and the optimization solution are 0.38 mm and 0.34 mm with the displacement of 10 mm. Then, when the displacement increases to 20 mm, the average reconstruction errors of the initial solution and the optimization solution are 0.79 mm and 0.73 mm. The error means in the initial method and the optimization method are 1.31 mm and 1.28 mm with the displacement of 30 mm. Finally, when the displacement grows to 40 mm, the average reconstruction errors in the initial solution and the optimization solution are 1.82 mm and 1.76 mm. The results describe that the optimization method contributes the higher accuracy than the initial solution generated from the non-homogeneous linear equations. The reconstruction errors of the initial solution and the optimization solution grow up as the displacements are on the rise. Furthermore, in the four groups of experiments, the average errors of the initial solution are 1.05 mm, 1.10 mm, 1.05 mm and 1.07 mm, respectively. While the average errors of the optimization solution are 0.98 mm, 1.05 mm, 1.00 mm and 1.03 mm, respectively. The optimization solutions are closer to the benchmarks than the initial solutions.

**Figure 4:**
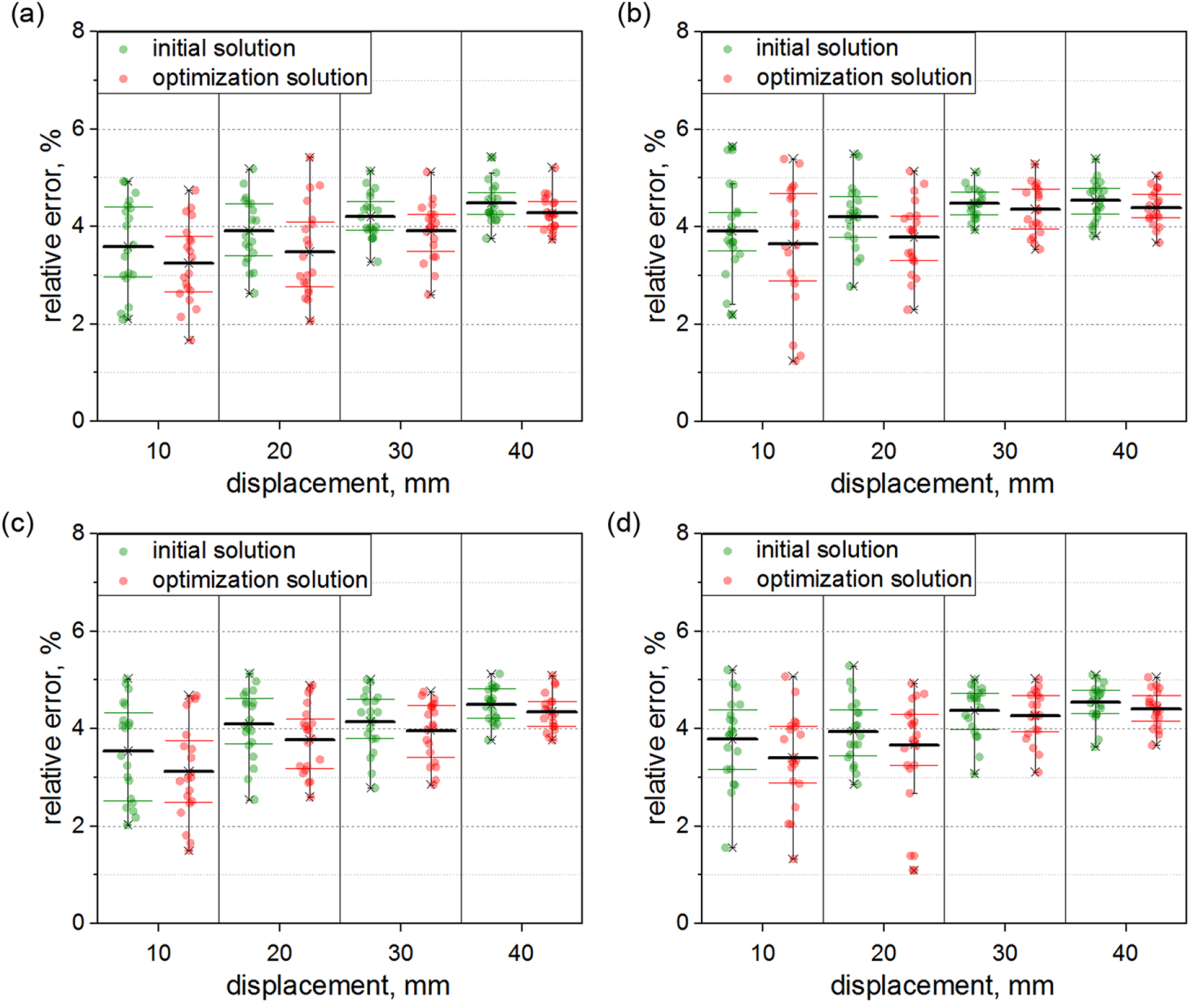
Relative errors of the reconstruction displacements, 10 mm, 20 mm, 30 mm, 40 mm. (**a**) The first group of experiments. (**b**) The second group of experiments. (**c**) The third group of experiments. (**d**) The fourth group of experiments.

The relative errors are illustrated in Fig. 4. In the first group of experiments, the averages of the relative errors of the initial solution and the optimization solution are 3.58% and 3.24% with the displacement of 10 mm. Then, when the displacement grows up to 20 mm, the mean relative errors of the initial solution and the optimization solution are 3.91% and 3.48%. The averages of the relative errors of the initial solution and the optimization solution are 4.20% and 3.91% with the displacement of 30 mm. Finally, when the displacement is 40 mm, the mean relative errors are 4.47% and 4.27%. In the second group of experiments, the averages of the relative errors of the initial solution and the optimization solution are 3.91% and 3.64% with the displacement of 10 mm. Then, when the displacement grows up to 20 mm, the mean relative errors of the initial solution and the optimization solution are 4.21% and 3.79%. The averages of the relative errors of the initial solution and the optimization solution are 4.47% and 4.35% with the displacement of 30 mm. Finally, when the displacement increases to 40 mm, the mean relative errors are 4.54% and 4.39%. In the third group of experiments, the averages of the relative errors of the initial solution and the optimization solution are 3.58% and 3.12% with the displacement of 10 mm. Then, when the displacement grows up to 20 mm, the mean relative errors of the initial solution and the optimization solution are 4.10% and 3.77%. The averages of the relative errors of the initial solution and the optimization solution are 4.14% and 3.96% with the displacement of 30 mm. Finally, when the displacement is 40 mm, the mean relative errors are 4.49% and 4.34%. In the fourth group of experiments, the averages of the relative errors of the initial solution and the optimization solution are 3.78% and 3.40% with the displacement of 10 mm. Then, when the displacement grows up to 20 mm, the mean relative errors of the initial solution and the optimization solution are 3.94% and 3.67%. The averages of the relative errors of the initial solution and the optimization solution are 4.37% and 4.27% with the displacement of 30 mm. Finally, when the displacement increases to 40 mm, the mean relative errors are 4.54% and 4.41%. The relative errors of the two methods slowly increase with the increasing displacement. In the four groups of experiments, the average relative errors of the initial solution are 4.04%, 4.28%, 4.08% and 4.16%, respectively. Then, the average relative errors of the optimization solution are 3.73%, 4.04%, 3.80% and 3.93%, respectively.

In the first group of experiments, when the displacement increases from 10 mm to 40 mm, the average reconstruction errors of the initial solution grow up from 0.36 mm to 1.79 mm. The average relative errors of the initial solution increase from 3.58% to 4.47%. The errors of the optimization solution grow up from 0.32 mm to 1.71 mm. The average relative errors of the optimization solution rise from 3.24% to 4.27%. In the second group of experiments, when the displacement increases from 10 mm to 40 mm, the average reconstruction errors of the initial solution grow up from 0.39 mm to 1.82 mm. The average relative errors of the initial solution increase from 3.91% to 4.54%. The errors of the optimization solution grow up from 0.36 mm to 1.75 mm. The average relative errors of the optimization solution rise from 3.64% to 4.39%. In the third group of experiments, when the displacement increases from 10 mm to 40 mm, the average reconstruction errors of the initial solution grow up from 0.35 mm to 1.80 mm. The average relative errors of the initial solution rise from 3.58% to 4.49%. The errors of the optimization solution grow up from 0.31 mm to 1.74 mm. The average relative errors of the optimization solution increase from 3.12% to 4.34%. In the fourth group of experiments, when the displacement increases from 10 mm to 40 mm, the average reconstruction errors of the initial solution grow up from 0.38 mm to 1.82 mm. The average relative errors of the initial solution rise from 3.78% to 4.54%. The errors of the optimization solution grow up from 0.34 mm to 1.76 mm. The average relative errors of the optimization solution increase from 3.40% to 4.41%. The relative errors of the optimization solution are smaller than the ones of the initial solution. In Fig. 3, the errors increase with the rising displacements from 10 mm to 40 mm. It is because the measurement depends on the trianglation of the camera and the orthogonal reference. The large errors correspond to the large side length. Therefore, the absolute errors increase with the grow-up displacement. However, in Fig. 4, the relative errors keep the similar level. It can be explained by Eq. (11). The benchmark displacement in the denominator increases with the rising absolute error in the numerator. Therefore, the growth trends of the relative errors are unobvious in Fig. 4.

**Figure 5:**
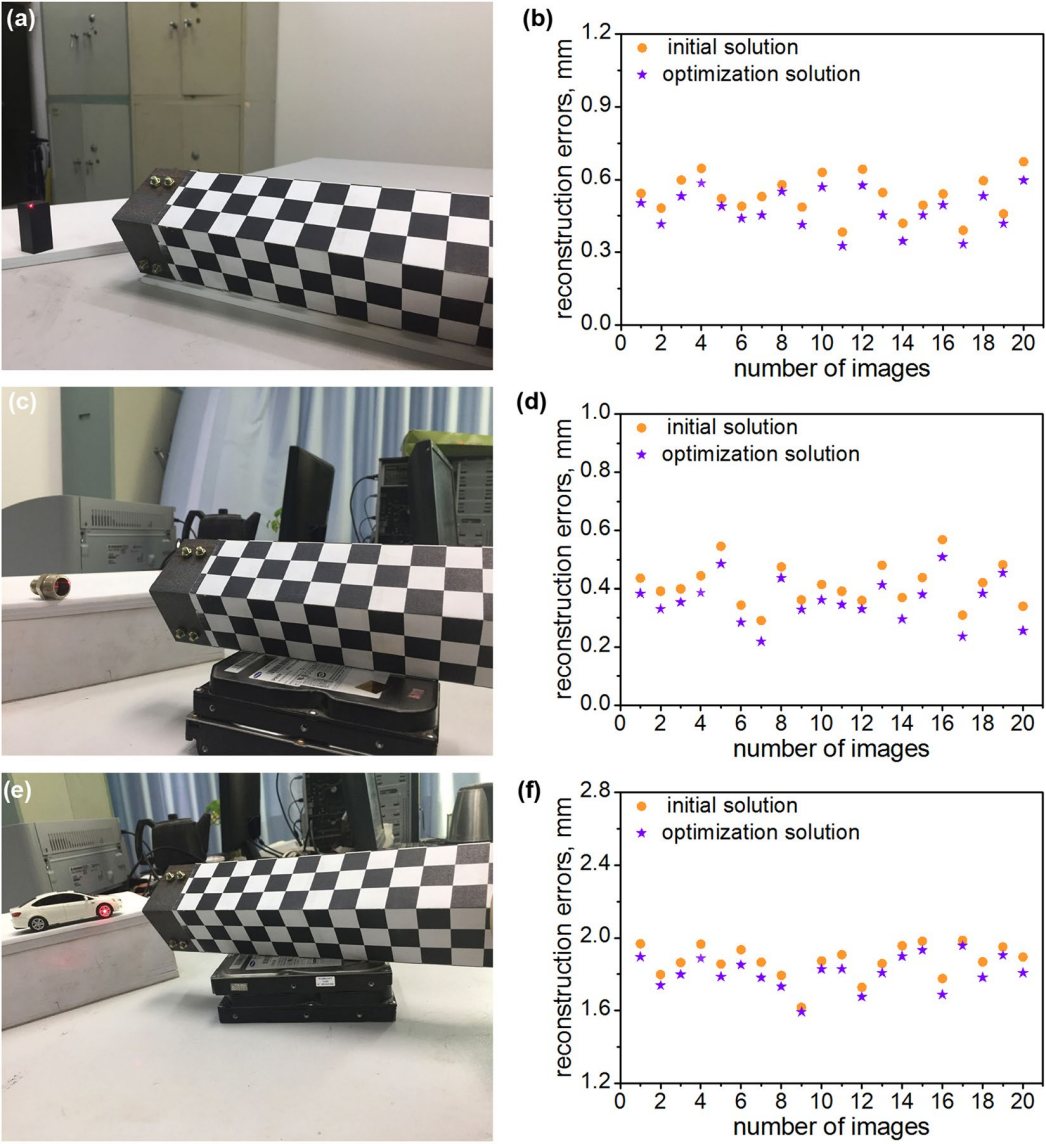
Three measurement cases of the reconstruction method adopting the projective point of the laser line coordinated by the orthogonal reference. (**a**) The experiment image of the cubic. (**b**) The reconstruction errors of the cubic. (**c**) The experiment image of the mechanical part. (**d**) The reconstruction errors of the mechanical part. (**e**) The experiment image of the car model. (**f**) The reconstruction errors of the car model.

Three cases are performed to show the practical utility of the reconstruction method. Three objects, including a cubic, a mechanical part, and a car model are measured to check the advantage of this method. The side length of the cubic, the diameter of the mechanical part and the wheelbase of the car model are reconstructed by the initial solution and the optimization solution of the method. The benchmark values of the measured dimensions, 19.25 mm, 15.56 mm and 63.32 mm, are derived from the vernier caliper. Every object is measured twenty times. The measurement results are shown in Fig. 5. The initial solution and the optimization solution are evaluated in the cases. The averages of the reconstruction errors are 0.53 mm, 0.41 mm and 1.87 mm, which are generated from the initial solutions. Furthermore, the averages of the reconstruction errors are 0.47 mm, 0.36 mm and 1.81 mm, which are generated from the optimization solutions. The measurement cases prove the application potentials of the reconstruction method.

The average absolute error of the initial solution is 1.07 mm while the one of the optimization solution is 1.01 mm. The initial solution provides a reasonable measurement, which is the beginning point of the optimization process. The advantage of solving the initial value is the simple calculation without the iteration. Therefore, the initial solution suits for the high-speed measurement. The optimization process spends time on the iterations to approach the optimization value. Thus, the optimization solution is valid for the high-accuracy measurement. The experiment results prove that the optimization method improves the reconstruction accuracy of the projective point of the laser line coordinated by the orthogonal reference. Furthermore, the experimental relative errors of the reconstruction method are lower than 5% that is the common accuracy of a measurement instrument.

## Discussion

An optimization method is proposed for the 3D reconstruction of the projective point of a laser line positioned by an orthogonal reference. The initial solution is provided by the non- homogeneous linear equations generated from the dual Plücker matrix of the laser line and then optimized by the parameterized re-projection errors. The reconstruction errors grow with the rising displacement. Moreover, the relative errors of the two methods are less than 5%. The optimization solution presents higher accuracy than the initial solution.

### Data availability

The datasets generated during the current study are available from the corresponding author on reasonable request.

## Electronic supplementary material

Supplementary Video S1
